# Conflict adaptation in a confound-minimized face-word Stroop task: exploring the potential settings of an fMRI-related experiment

**DOI:** 10.3389/fpsyg.2026.1794231

**Published:** 2026-06-15

**Authors:** Leonardo M. Caisachana Guevara, Robert Huber, Harun Kocataş, Mohamed Abdelmotaleb, Marcus Meinzer, Rico Fischer

**Affiliations:** 1 Department of Psychology, University of Greifswald, Greifswald, Germany; 2 Department of Neurology, University Medicine Greifswald, Greifswald, Germany

**Keywords:** conflict, conflict adaptation, congruence sequence effect, fMRI, Stroop

## Abstract

Conflict adaptation refers to the adjustment of cognitive control to reduce interference on subsequent trials. Its neural mechanisms are often studied by assessing the congruence sequence effect (CSE) in face–word Stroop tasks during neuroimaging. The present behavioral study aimed to address two key methodological issues when aiming to translate standard response conflict tasks into fMRI settings: Ensuring the involvement of cognitive control in the CSE and demonstrating its temporal stability across varying inter-trial intervals. In a set of five experiments, using an adapted version of the face–word Stroop task, participants performed either a gender categorization (Experiment 3) or responded to the learned identity of four faces while ignoring the printed name (all other experiments). We specifically incorporated recent advancements in confound minimization to exclude effects of feature integration and contingency learning, included a priming component to further increase the involvement of cognitive control, and used a variable range of inter-trial intervals to test for potential applicability in fMRI research. Although a robust Stroop effect was consistently observed in all experiments, a strong CSE emerged specifically in the gender categorization task. In the confound-minimized face–word Stroop variant, a significant CSE occurred only under specific conditions (e.g., sufficiently long target presentation, bi-manual responses) and was accompanied by substantial inter-individual variability. Therefore, while we observed a reliable CSE in a confound-minimized face–word Stroop task with long inter-trial intervals, the increased methodological rigor that improves precision and interpretability also adds complexity and yields smaller and more variable effects. This trade-off constitutes an important consideration when aiming to adapt the Stroop task for fMRI applicability.

## Introduction

Cognitive control enables humans to adapt their behavior flexibly to situational demands. The conflict-monitoring theory (CMT), proposed by Botvinick et al. (2001), describes a computational framework for understanding how cognitive control is recruited to adjust thoughts and actions to changing environmental requirements. It emphasizes a functional role of response conflict in signaling the need for control engagement. Response conflicts arise when stimuli contain both task-relevant and task-irrelevant features. A widely used experimental paradigm to study conflict and conflict resolution is the Stroop task (Stroop, 1935; see MacLeod, 1991, for a review), in which participants typically respond to the ink color of a color word. Response times (RT) and error rates (ER) increase when the print color mismatches the color word in incongruent trials (e.g., RED printed in green) compared to congruent trials when the print color matches the color word (e.g., RED printed in red). The CMT proposes an outcome evaluation system, presumably located in the anterior cingulate cortex (ACC), dedicated to detecting such response conflicts and providing triggers for control adjustments via the dorsolateral prefrontal cortex (DLPFC) (Botvinick et al., 2004). The detection of a response conflict in an incongruent Stroop trial would thus serve as a signal to recruit cognitive control to optimize performance in a subsequent trial. A prominent behavioral marker of this regulation is the congruence sequence effect (CSE), which describes a reduction of the congruency effect (i.e., the performance difference between incongruent and congruent trials) following an incongruent trial compared to a congruent trial (Gratton et al., 1992; for reviews, see Duthoo et al., 2014a; Egner, 2007, 2017).

Consistent with the idea of conflict detection and trial-by-trial adjustments in cognitive control, Kerns et al. (2004) found larger ACC activation for incongruent trials that followed non-conflict congruent trials (cI transitions) compared to incongruent trials that were preceded by incongruent trials (iI transitions). This pattern suggests that the heightened ACC activity in cI transitions reflects increased neural demands for conflict detection, whereas in iI transitions, conflict-induced control recruitment in trial N-1 reduces the subsequent experience of conflict in trial N. Furthermore, the magnitude of the ACC activation in trial N-1 predicted the degree of DLPFC recruitment on the following trial N. Subsequent seminal imaging studies have provided insights into the neural mechanisms of adaptive control. Egner and Hirsch (2005), for example, used a face-word Stroop task and showed that conflict in trial N-1 elicited increased functional connectivity between the DLPFC and task-relevant sensory regions, namely the fusiform face area, FFA. These findings suggest that conflict enhances the selective amplification of task-relevant information rather than inhibiting task-irrelevant information.

Taken together, the CMT provides a formal computational account of adaptive cognitive control. The detection of conflict indicates when top-down engagement is required, and the magnitude of conflict determines how regulatory resources are allocated. CMT thus explains flexible, trial-by-trial control adjustments without invoking a central executive or “homunculus.” Since its original proposal, the theory has been consistently refined and extended (Botvinick, 2007; Egner, 2008; Verguts and Notebaert, 2009; Hazeltine et al., 2011; Dreisbach and Fischer, 2015), and unsurprisingly, has stimulated extensive research over the last 25 years in both cognitive psychology and cognitive neuroscience (see Lee et al., 2025, for a meta-analysis).

Despite the theoretical and empirical success of the CMT, the application of standard response-conflict paradigms to investigate CSEs in fMRI research remains problematic and faces at least two key methodological challenges. First, it must be ensured that an observed CSE can be attributed to cognitive control to be interpreted as evidence for conflict adaptation (see Braem et al., 2019; Egner, 2017, for an overview). Second, because the CSE reflects a trial-by-trial adjustment, its measurement may be constrained by temporal factors that are at odds with the temporal jittering necessary for accurately assessing the BOLD signal in fMRI studies.

The question of the extent to which a CSE can be interpreted by factors other than cognitive control adjustments has been a notorious and controversial debate. Especially, the use of small stimulus and response sets can increase the likelihood of feature integration effects (Mayr et al., 2003; Hommel and Colzato, 2004) and contingency learning confounds (Schmidt et al., 2007; Schmidt and De Houwer, 2011) that produce a CSE pattern devoid of any control assumptions (see Egner, 2017, for an overview). Hence, refinements and recommendations for experimental paradigms have been postulated to ensure an unequivocal interpretation of the CSE as a control-based measure of conflict adaptation. For instance, confound-minimized designs eliminate feature repetitions and balance contingencies, thereby avoiding feature integration and contingency learning mechanisms. To achieve this, researchers have implemented two alternating 2-alternative forced-choice (AFC) tasks, with separate stimulus and response sets (e.g., Kim and Cho, 2014; Weissman et al., 2015b; Weissman, 2020). In a Stroop task, for example, a confound-minimized 4-AFC task could alternate between a red/blue stimulus–response pair and a yellow/green stimulus–response pair from one trial to the next. This precludes any stimulus- and response repetitions. In addition, incongruent trials exclusively consist of stimulus combinations within each stimulus–response pair (e.g., RED printed in blue) but not across pairs (e.g., RED printed in green). This ensures an equal distribution of unique congruent and incongruent stimuli within each stimulus set, thereby avoiding potential contingency learning confounds (e.g., Schmidt and Weissman, 2014). Although this approach increases the complexity of CSE assessment, it helps to minimize influences that are unrelated to cognitive control (Braem et al., 2019). Although the Stroop task remains highly relevant and widely used in neuroimaging studies for investigating conflict (see Müller et al., 2025, for a meta-analysis) and cognitive control adjustments by assessing the CSE (Hinault et al., 2018; Kar et al., 2019; Lee and Kim, 2019; Teubner-Rhodes et al., 2019; Yang et al., 2022; Kaminski et al., 2023; Oren et al., 2023; Wang et al., 2023; Xu et al., 2024), we are not aware of fMRI studies using a Stroop task that have employed confound-minimized designs as recommended in a recent consensus paper (Braem et al., 2019). Thus, it remains unclear to what extent early findings of seminal fMRI studies on conflict adaptation can be replicated when using more methodologically advanced setups of confound-minimized Stroop tasks.

The second methodological challenge regards the temporal requirements for obtaining a reliable BOLD response in fMRI studies. Specifically, to avoid hemodynamic overlap and reduce regressor collinearity, jittered inter-trial intervals (ITIs) between 2 and 6 s are recommended (Burock et al., 1998). A substantial body of fMRI studies has reported reliable CSEs when employing neuroimaging-optimized designs with relatively long inter-trial intervals. However, direct investigations of the temporal characteristics of CSEs in a Stroop task are scarce. Findings of a face-word Stroop task showed a pronounced decay of the CSE when inter-stimulus or response–stimulus intervals exceeded 2,500–3,000 ms (Egner et al., 2010; see also Duthoo et al., 2014b, Experiment 1), even when using a paradigm closely related to the one successfully applied in fMRI research (Egner and Hirsch, 2005; Egner et al., 2007, 2008). This rapid decay might be directly related to the temporal characteristics of stimulus–response bindings, which are present in non-confound-minimized conflict tasks (Schiltenwolf et al., 2023). To rule out binding effects contributing to the CSE, Schiltenwolf et al. (2023) tested the temporal stability of the CSE by implementing a confound-minimized design. Namely, two subsets of stimuli and corresponding responses alternated on consecutive trials, thereby preventing stimulus and response repetitions as well as negative priming effects. Each stimulus occurred equally often in congruent and incongruent conditions, thus preventing contingency learning. The two response sets were mapped on the four fingers of the right hand (excluding the thumb). However, it remains to be determined to what extent the temporal properties of the CSE observed in a number-priming task (Schiltenwolf et al., 2023) generalize to CSEs obtained in a face–word Stroop task.

The present study aimed to replicate early seminal findings on the adjustments of cognitive control in a face-word Stroop paradigm (Egner and Hirsch, 2005). We focused on the face-word Stroop paradigm because, particularly in neuroimaging research, the dissociation of sensory regions processing task-relevant (face) and task-irrelevant (word) information allows for a more precise characterization of how cognitive control operates in order to solve and adjust to conflict, i.e., by strengthening task-relevant sensory processing and/or reducing task-irrelevant sensory processing (see Egner and Hirsch, 2005, and in analogy Stelzel et al., 2009, for a further description of this logic). We explicitly incorporated recent advancements in confound minimization to exclude effects of feature integration and contingency learning. To examine their suitability for fMRI research, we also employed a variable range of inter-trial intervals. An overview of the main parameters in all five experiments is shown in Table 1.

**Table 1:** Overview of the main parameters of the conducted experiments (Exp) in this study.

**Exp**	***N***	**Target faces**	**Target duration**	**Responses**	**ITI**	**Task**	**Stroop** **effect** **in ms**	**CSE in ms (SD)**
1	42	4 unknown faces	200 ms	Right hand: IF-MF-RF-LF	1, 1.5, 2, 2.5, 3, 3.5, 4, 5 s	Two 2-AFC online	48***	8.4 (53.1)
2	51	4 celebrities	200 ms	Right hand: IF-MF-RF-LF	1, 1.5, 2, 2.5, 3, 3.5, 4, 5 s	Two 2-AFC online	53***	7.6 (45.7)
3	40	4 unknown faces	1,000 ms	Two hands: IF / IF	1, 1.5, 2, 2.5, 3, 3.5, 4, 5 s	One 2-AFC lab	54***	20.5* (22.0)
4a	40	4 unknown faces	1,000 ms	Two hands: MF-IF / IF-MF	1, 1.5, 2, 2.5, 3, 3.5, 4, 5 s	Two 2-AFC online	56***	16.4* (39.9)
4b	40	4 unknown faces	1,000 ms	Two hands: MF-IF / IF-MF	2, 2.5, 3, 3.5, 4, 5 s	Two 2-AFC online	90***	12.6* (35.5)

N – number of participants, IF – index finger, MF – middle finger, RF – ring finger, LF – little finger, 2-AFC – two-alternative forced-choice task, CSE – congruence sequence effect. *p < 0.05, ***p < 0.001.

## Experiment 1

In Experiment 1, participants performed an adapted version of the face-word Stroop task. First, to ensure that the measured CSE can be attributed to cognitive control adjustments, the classical face-word Stroop task was modified to meet the requirements of a confound-minimized design. More precisely, participants did not make binary categorical decisions (e.g., sex, age, emotional expression). Instead, they responded to the identity of one of four faces, each mapped to a distinct response key, while ignoring the person’s name, which was overlaid on the face. To enable this, participants first learned the associations between four alternatives: names, their corresponding faces, and the required response. As in Schiltenwolf et al. (2023), all responses were made with the fingers of the right hand. Second, we also included a priming component to increase response conflict (Wendt et al., 2014) and to strengthen further the involvement of cognitive control in the CSE (Weissman et al., 2014; Weissman et al., 2015b). Finally, we tested whether a reliable CSE can be obtained when long and variable inter-trial intervals were included.

### Methods

#### Participants

The seminal study by Egner and Hirsch (2005) reported an effect size η_p_^2^ = 0.242 for the CSE in the face-target condition based on a sample of 22 participants. Because of the confound-minimized design in the present study, we decided to test at least 40 participants in each experiment. No *a priori* power analysis was conducted. To evaluate the sensitivity of the present 2 × 2 within-subjects design of Experiments 1–4, a post-hoc sensitivity analysis was conducted using GPower (Faul et al., 2007). Given a minimum sample size of *N* = 40 in the present experiments, an *α*-level of 0.05, and a desired power of 1-*β* = 0.80, the analysis indicated that the design of our experiments was sufficiently powered to detect interaction effects of at least *f* = 0.188, corresponding to a ηp^2^ of approximately 0.034. A total of 43 students from the University of Greifswald (31 female; *M*_age_ = 25.7 years, SD = 7.7 years, range: 18–59 years) participated in the study. All participants received course credits or €5. All participants had normal or corrected-to-normal vision, and all but seven reported being right-handed. None of the participants had a first name that was used as a stimulus in the study. Participants provided informed consent before their inclusion in the study in accordance with the ethical standards of the 1964 Declaration of Helsinki and of the German Psychological Society.

#### Stimuli and apparatus

Pictures of two male and two female faces from the Radboud Faces Database (Langner et al., 2010) served as target stimuli. They were presented in the center of the screen, scaled relative to the size of the participant’s screen (i.e., picture width and height were 30%/45% of the screen’s size, respectively). Word stimuli consisted of two male (i.e., Jonas, Lukas) and two female (i.e., Clara, Hanna) five-letter names that were presented in capital 24-point Arial font in white in the center of the screen. A white plus sign in 24-point Arial font displayed in the screen’s center served as a fixation cross. Responses were made with the right hand using the index, middle, ring, and little fingers (V, B, N, or M on a QWERTZ keyboard). PsychoPy (Peirce et al., 2019) was used for stimulus presentation and data recording.

#### Procedure

The study was programmed in PsychoPy (Peirce et al., 2019) and conducted online (Bridges et al., 2020) using Pavlovia.^1^ In the experiment, participants were required to respond based on the identity of four different face stimuli while ignoring the names printed on the faces. Before the experiment, participants completed a learning phase to acquire the identities of the individuals and their corresponding response buttons. An instruction screen introduced the face, the name, and the response key of each person. Subsequently, they performed three training blocks (20 trials each) in a fixed order. Participants responded first to the identity of the face stimuli (Block 1), then to the name stimuli (Block 2), and finally to the face of a person, with the corresponding (congruent) name printed on top (Block 3). Stimuli remained on the screen until a response was executed. Feedback for incorrect responses was delivered for a duration of 300 ms. The instruction screen with the stimulus–response assignments was presented as a reminder after each block. Training blocks were repeated if the accuracy was below 80%.

**Figure 1:**
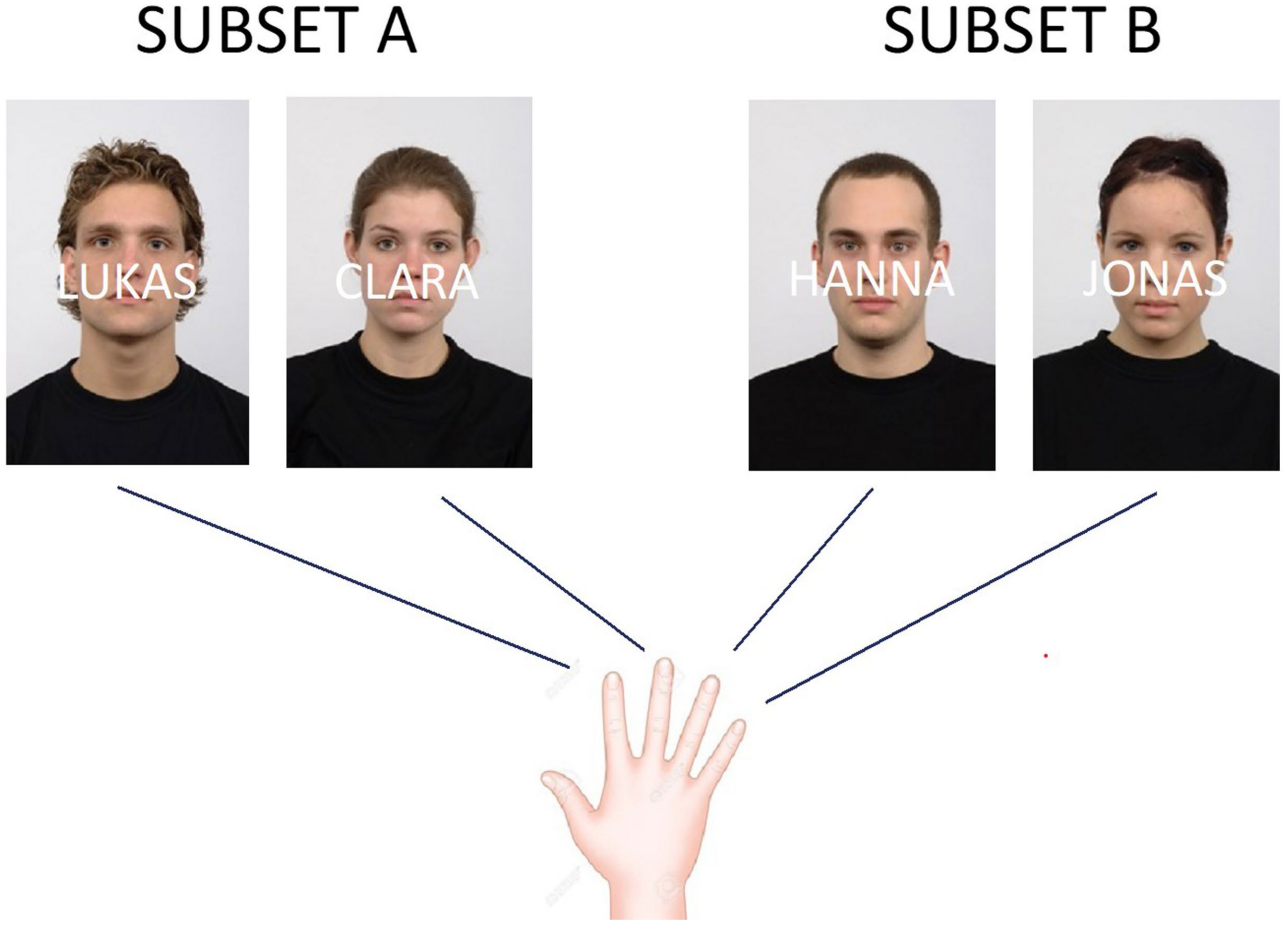
Two face and two name stimuli formed a subset of stimuli and responses. Each face-name identity was mapped on a distinct response. In the learning phase of Experiment 1, names, faces, or congruent face-name combinations were presented in separate blocks. In the experiment, face-name combinations were congruent and incongruent. Congruent face-name pairings are shown for Subset A and incongruent face-name pairings are shown for Subset B. Incongruent face-name pairing occurred only within each subset. Stimuli and responses of Subsets A and B alternated on a trial-by-trial basis. The four response alternatives were mapped onto the fingers of the right hand (Schiltenwolf et al., 2023).

The subsequent phase consisted of two 2-alternative forced-choice tasks. That is, participants were informed about two subsets of stimuli that were paired with their distinct practiced responses (see Figure 1). Each subset contained a specific male and a female face stimulus, i.e., Lukas and Clara (Subset A) and Jonas and Hanna (Subset B). Half of the participants responded to Lukas and Clara (Subset A) with the right index and middle finger and to Jonas and Hanna (Subset B) with the right ring and little finger, respectively. The other half responded to Jonas and Hanna (Subset B) with the right index and middle finger and to Lukas and Clara (Subset A) with the right ring and little finger, respectively.

Participants were instructed to respond to the identity of the face stimulus as fast and accurately as possible by pressing the response key assigned to the name of the person (e.g., face of Lukas – right index finger). They were further instructed to ignore the name printed overlaid on the face. A congruent trial consisted of a matching face and name (e.g., the name Lukas printed on Lukas’ face). An incongruent trial consisted of a mismatching face-name combination from each subset (e.g., the name Clara printed on Lukas’ face or vice versa). An incongruent pairing across the two subsets of stimuli (e.g., the name Hanna printed on Lukas’ face) was not possible. Each stimulus served equally often in congruent and incongruent trials. Stimuli of Subset A and Subset B alternated on a trial-by-trial basis, avoiding any target, distracter, and response repetitions.

A trial started with the presentation of the prime word for 200 ms. This prime stimulus was followed by a blank screen for 50 ms. The target face stimulus was presented for 200 ms, after which the screen remained black until either a response or a maximum time of 2050 ms occurred. Correct answers were followed by a blank screen for 300 ms. Alternatively, feedback consisted of the German words “falsch” (wrong) or “zu langsam” (too slow) for 300 ms. A fixation sign was presented during the variable inter-trial interval (i.e., 1,000, 1,500, 2000, 2,500, 3,000, 3,500, 4,000, and 5,000 ms).

The experimental phase began with 16 practice trials, followed by five blocks of 64 trials each, for a total of 320 experimental trials. The learning and experimental phases lasted approximately 30 min.

#### Design

A 2 (Congruence_N_: congruent vs. incongruent) × 2 (Congruence_N-1_: congruent vs. incongruent) within-subjects repeated measures design was applied.

### Results

#### Data analysis

**Figure 2:**
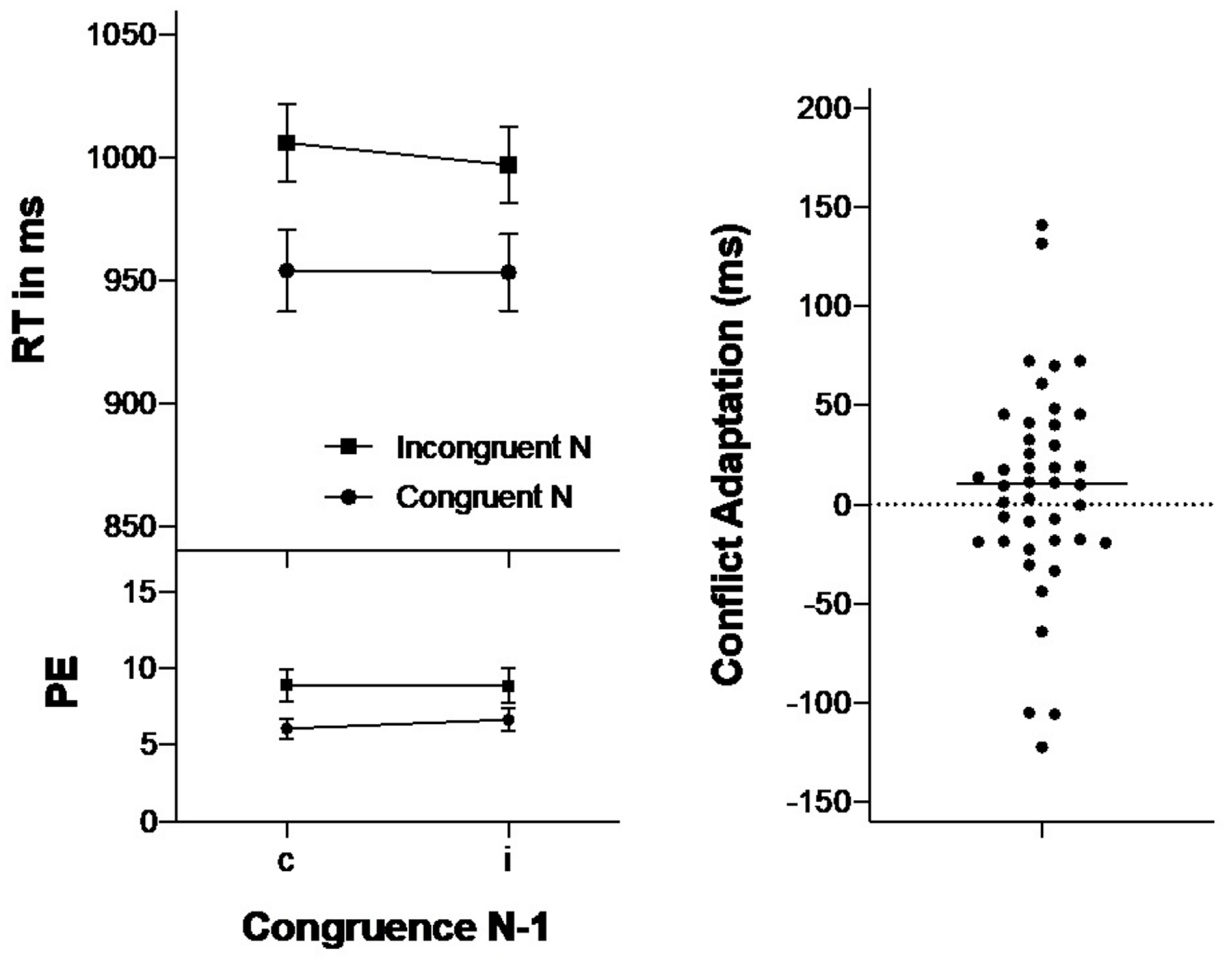
Response times (RT) and percent error (PE) in Experiment 1 are presented as a function of previous and current congruency (left). The individual congruence sequence effects (CSE) are calculated as the congruence effect following congruent trials minus the congruence effect following incongruent trials. The sample mean is represented by the horizontal solid line (right). Error bars represent standard errors of the mean.

One participant was excluded before analyses due to unusually high error rates (>35%). The repeated measures ANOVA, with the within-subject factors Congruence_N_ and Congruence_N-1,_ was conducted on RTs and percent error (PE). For all analyses, the first trial of each block, post-error trials, and trials in which RTs exceeded the response deadline (missed trials) were excluded (11.1%). For the RT analysis, incorrect trials, (7.6%) and trials with RTs smaller or larger than 3 SD of the individual condition mean (1.5%) were removed. One-sided paired t-tests were run to test for the influence of previous (non)conflict trials on current incongruent trials and on current congruent trials. Results are presented in Figure 2.

#### RT

Results revealed a strong congruence effect, *F*(1, 41) = 91.20, *p* < 0.001, η_p_^2^ = 0.69, with faster responses in congruent (954 ms) than in incongruent (1,002 ms) trials. There was no effect of previous congruence, *F*(1, 41) = 2.75, *p* = 0.105, η_p_^2^ = 0.06. Similarly, the factors Congruence_N-1_ and Congruence_N_ did not interact, *F*(1, 41) = 1.04, *p* = 0.313, η_p_^2^ = 0.03. The result did not change when restricting the analysis to right-handed persons only, *F* < 1. Despite an absent overall CSE, previous conflict (compared to previous non-conflict) reduced RTs in subsequent incongruent trials, *t*(41) = 2.02, *p* = 0.025 (one-sided). The analogous slowing for congruent trials following conflict was not observed, *t*(41) = 0.14, *p* = 0.455 (one-sided). A follow-up exploratory analysis with the additional 8-level factor Inter-trial-interval did not yield a three-way interaction between Inter-trial-interval, Congruence_N_, and Congruence_N-1_, *F*(7, 287) = 0.79, *p* = 0.599, η_p_^2^ = 0.02.

#### PE

The repeated measures ANOVA on error rates closely mirrored the RT results. There was only a significant effect of Congruence_N_, *F*(1, 41) = 8.93, *p* = 0.005, η_p_^2^ = 0.18, with fewer errors in congruent (6.4%) than in incongruent trials (8.9%). As in RTs, Congruence_N-1_ and Congruence_N_ did not interact, *F* < 1. Subsequent t-tests confirmed this finding, both *t*’s < 1.

### Discussion

Experiment 1 served to test for a CSE in a face-word Stroop task using a wide range of different inter-trial intervals. Surprisingly, a statistically significant CSE was not observed. Yet, incongruent trial RTs were significantly reduced after conflict compared to following non-conflict trials, as predicted by the conflict monitoring model.

## Experiment 2

Because Experiment 1 did not yield the expected CSE, we aimed to replicate Experiment 1, seeking potentially stronger CSEs. For this, we decided to run a version with celebrities as target stimuli in Experiment 2, because previous studies using a face-word Stroop task with famous faces (actors and politicians) reported a substantial modulation of congruence effects by previous conflict with inter-trial intervals varying between 3 and 5 s (Egner and Hirsch, 2005). However, previous studies did not use a confound-minimized experimental design to study CSEs.

### Methods

#### Participants

Fifty-two students and members from the University of Greifswald (36 female; *M*_age_ = 24.8 years, SD = 8.3 years, range 18–61 years) participated in the online experiment. All participants received course credits or €5. All participants had normal or corrected-to-normal vision, and all but one reported being right-handed. Participants provided informed consent before their inclusion in the study in accordance with the ethical standards of the 1964 Declaration of Helsinki and of the German Psychological Society.

#### Stimuli and apparatus

Target stimuli contained portraits of two male (Daniel Radcliffe, Johnny Depp) and two female (Angelina Jolie, Jennifer Lawrence) celebrities. Prime stimuli and distracter words consisted of the full names of the celebrities. Response fingers and response keys were identical to Experiment 1.

#### Procedure

The same variable inter-trial intervals (i.e., 1,000, 1,500, 2000, 2,500, 3,000, 3,500, 4,000, and 5,000 ms) were included as in Experiment 1. Analogously to Experiment 1, two face and two name stimuli formed trial-wise alternating subsets of stimuli and responses (i.e., Johnny Depp and Angelina Jolie versus Daniel Radcliffe and Jennifer Lawrence). The word distracter consisted of the celebrity’s full name. The assignment of each subset of stimuli (index and middle finger or ring and little finger) was counterbalanced across participants. The same learning phase was applied as in Experiment 1.

#### Design

A 2 (Congruence_N_: congruent vs. incongruent) × 2 (Congruence_N-1_: congruent vs. incongruent) within-subjects repeated measures design was applied.

### Results

#### Data analysis

**Figure 3:**
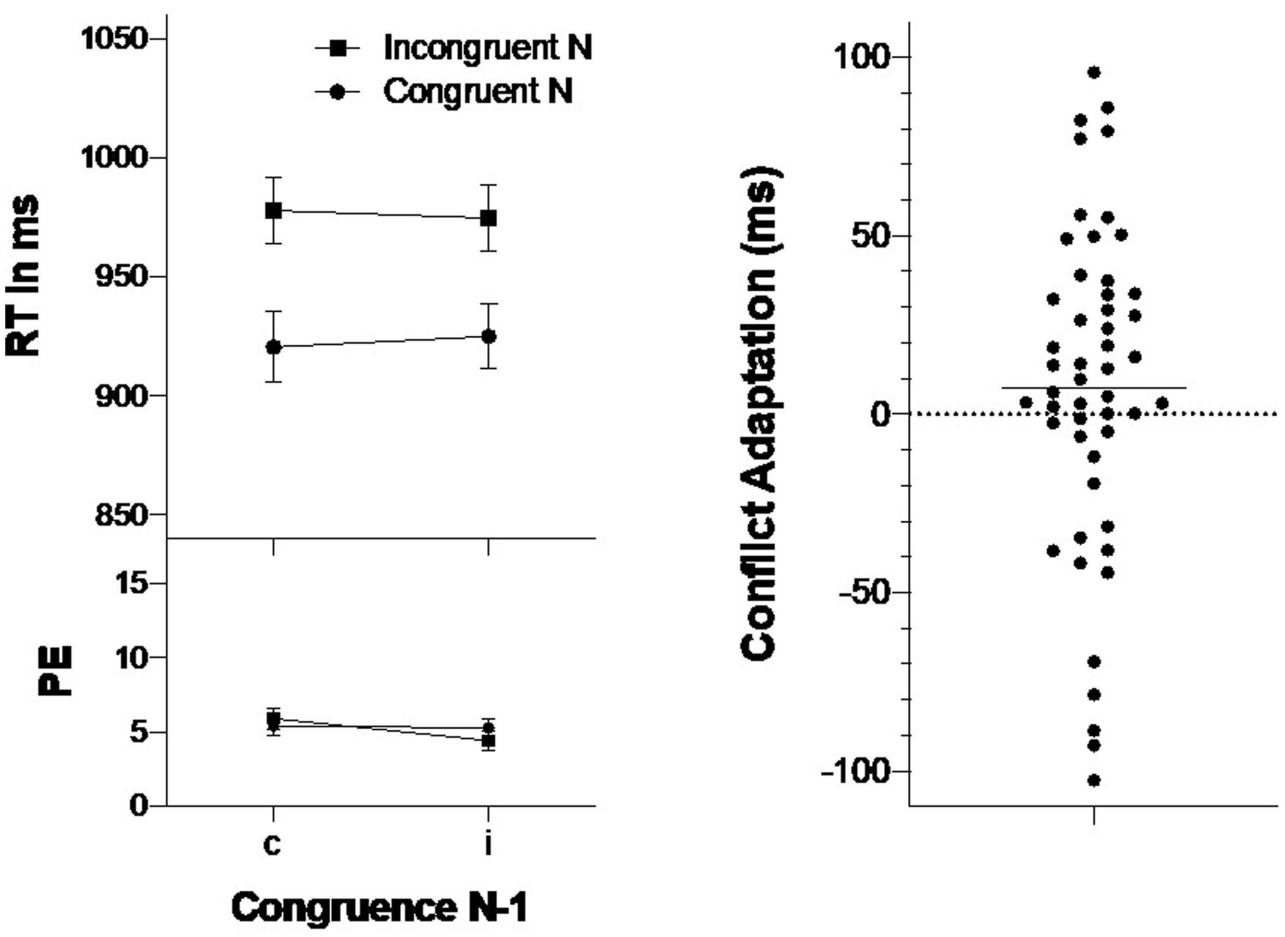
Response times (RT) and percent error (PE) in Experiment 2 using celebrities as target stimuli are presented as a function of previous and current congruency (left). The individual congruence sequence effects (CSE) are calculated as the congruence effect following congruent trials minus the congruence effect following incongruent trials. The sample mean is represented by the horizontal solid line (right). Error bars represent standard errors of the mean.

One participant was excluded before analyses due to unusually high error rates (>42%). The repeated measures ANOVA with the within-subject factors Congruence_N_ and Congruence_N-1_ was conducted on RTs and percent error (PE). For all analyses, the first trial of each block, post-error trials, and trials in which RTs exceeded the response deadline (missed trials) were excluded (8.0%). For the RT analysis, incorrect trials (5.3%) and trials with RTs smaller or larger than 3 SD of the individual condition mean (1.6%) were removed. One-sided paired *t*-tests were run to test for the influence of previous (non)conflict trials on current incongruent trials and on current congruent trials. Results are presented in Figure 3.

#### RT

Results again demonstrated a large congruence effect, *F*(1, 50) = 119.10, *p* < 0.001, η_p_^2^ = 0.70. Participants responded faster in congruent (923 ms) than in incongruent (976 ms) trials. The effect of previous congruence was not significant, *F*(1, 50) = 0.36, *p* = 0.850, η_p_^2^ = 0.00. Also, the factors Congruence_N-1_ and Congruence_N_ did not interact, *F*(1, 50) = 1.40, *p* = 0.242, η_p_^2^ = 0.03. The result did not change when restricting the analysis to right-handed persons only, *F*(1, 49) = 1.44, *p* = 0.236, ηp^2^ = 0.03, when excluding participants who reported not being familiar with at least one of the celebrities, *F*(1, 42) = 1.20, *p* = 0.279, ηp^2^ = 0.03, or when excluding two participants with > 50 years of age, *F*(1, 48) = 1.74, *p* = 0.194, ηp^2^ = 0.04. For reasons of completeness, subsequent *t*-tests were conducted. However, neither RTs in incongruent trials, *t*(50) = 0.63, *p* = 0.265 (one-sided), nor RTs in congruent trials, *t*(50) = −1.04, *p* = 0.153 (one-sided) were affected by previous conflict (compared to previous non-conflict). A follow-up exploratory analysis with the additional 8-level factor Inter-trial-interval did not yield a three-way interaction between Inter-trial-interval, Congruence_N_, and Congruence_N-1_, *F*(7, 350) = 1.66, *p* = 0.118, η_p_^2^ = 0.03.

#### PE

The repeated measures ANOVA on error rates revealed a significant main effect of Congruence_N-1_, *F*(1, 50) = 4.67, *p* = 0.036, η_p_^2^ = 0.09. Slightly fewer errors were committed following conflict (4.9%) than following non-conflict trials (5.7%). There was no effect of Congruence_N_, *F*(1, 50) = 0.15, *p* = 0.705, η_p_^2^ = 0.00, and the interaction between the two factors missed the level of statistical significance, *F*(1, 50) = 3.49, *p* = 0.068, η_p_^2^ = 0.07. Subsequent t-tests showed that previous conflict (compared to previous non-conflict) reduced the error rates in subsequent incongruent trials, *t*(50) = 3.02, *p* = 0.002 (one-sided), but did not affect error rates in subsequent congruent trials, *t*(50) = 0.27, *p* = 0.395 (one-sided).

#### Between-experiment comparison

Since Experiments 1 and 2 were largely identical, except for the use of unknown versus known faces, and because the results of both experiments were inconclusive, an analysis of the combined data set with N = 93 was conducted to increase statistical power, including Experiment as a between-subject factor. However, the interaction between Congruence_N-1_ and Congruence_N_ did not become significant in RTs, *F*(1, 91) = 2.42, *p* = 0.123, η_p_^2^ = 0.03, or in error rates, *F*(1, 91) = 2.73, *p* = 0.102, η_p_^2^ = 0.03.

### Discussion

The strategy to use familiar faces of celebrities in Experiment 2 did not increase the size of the CSE as expected. In error rates, but not RTs, a CSE only approached the level of statistical significance. Fewer errors were observed in incongruent trials when following conflict compared to non-conflict trials in N-1. Although eight participants reported not being familiar with at least one of the celebrities, Experiment 2 contained the same training part to establish the stimulus–response mapping as in Experiment 1. In addition, excluding these eight participants from the analyses did not alter the results. Thus, unfamiliarity with the celebrities does not seem to underlie the missing CSE. In addition, an analysis of the combined data from both experiments did not reveal a significant CSE in either RTs error rates.

## Experiment 3

Using a confound-minimized design entails increased task complexity, for example, requiring four responses instead of two. In addition, finger movement control might differ considerably between the two subsets of right-hand responses (index-middle vs. ring-little) in Experiments 1 and 2. Given the unexpected difficulty in obtaining a CSE during our first two experiments, we aimed to optimize the experimental design in order to obtain a reliable CSE. For this, Experiment 3 was simplified into a conventional binary choice conflict task, in which participants categorized the same stimulus material from Experiment 1 as male or female by responding with the left or right index finger, respectively. It has been shown that designs that allow for bottom-up confounds and contain smaller stimulus–response sets result in larger CSEs than confound-minimized designs (e.g., Blais and Verguts, 2012; Lee et al., 2025). Additionally, stimulus duration was increased to 1,000 ms (Egner and Hirsch, 2005), and all participants were tested in person in the lab.

### Methods

#### Participants

Forty participants from the University of Greifswald (25 female, 1 diverse; *M*_age_ = 23.0 years, SD = 4.2 years, range 18–34 years) participated in the experiment and received either course credits or €5. All participants had normal or corrected-to-normal vision, and all but three reported being right-handed, including one ambidextrous participant. Participants provided informed consent before their inclusion in the study in accordance with the ethical standards of the 1964 Declaration of Helsinki and of the German Psychological Society.

#### Stimuli and apparatus

The same face stimuli from Experiment 1 were employed. The German words for “male” (Mann) and “female” (Frau) served as prime/distracter stimulus. Participants responded with their left and right index fingers on the X and M keys of a QWERTZ keyboard, respectively.

#### Procedure

The procedure was identical to Experiment 1, with the following exceptions: The prime word was presented for 200 ms and was followed immediately by the onset of the target stimulus. The target stimulus duration was increased until a response execution or for a maximum of 1,000 ms. Responses given after 1800 ms of target stimulus onset were considered as misses. To avoid identical prime/distracter repetitions, words were presented either in small or capitalized letters on a trial-by-trial basis. Because the task required gender differentiation, no learning phase was included. Half of the participants categorized face stimuli as female with their left index finger and as male with their right index finger. This mapping was reversed for the other half of the participants. In addition, since the same stimulus subsets as in Experiment 1 also alternated from trial to trial, the pairings of stimulus sets and lowercase versus uppercase prime/distracter letters were counterbalanced across participants. The experiment included eight practice trials and five experimental blocks of 64 trials each.

#### Design

A 2 (Congruence_N_: congruent vs. incongruent) × 2 (Congruence_N-1_: congruent vs. incongruent) within-subjects repeated measures design was applied.

### Results

#### Data analysis

**Figure 4:**
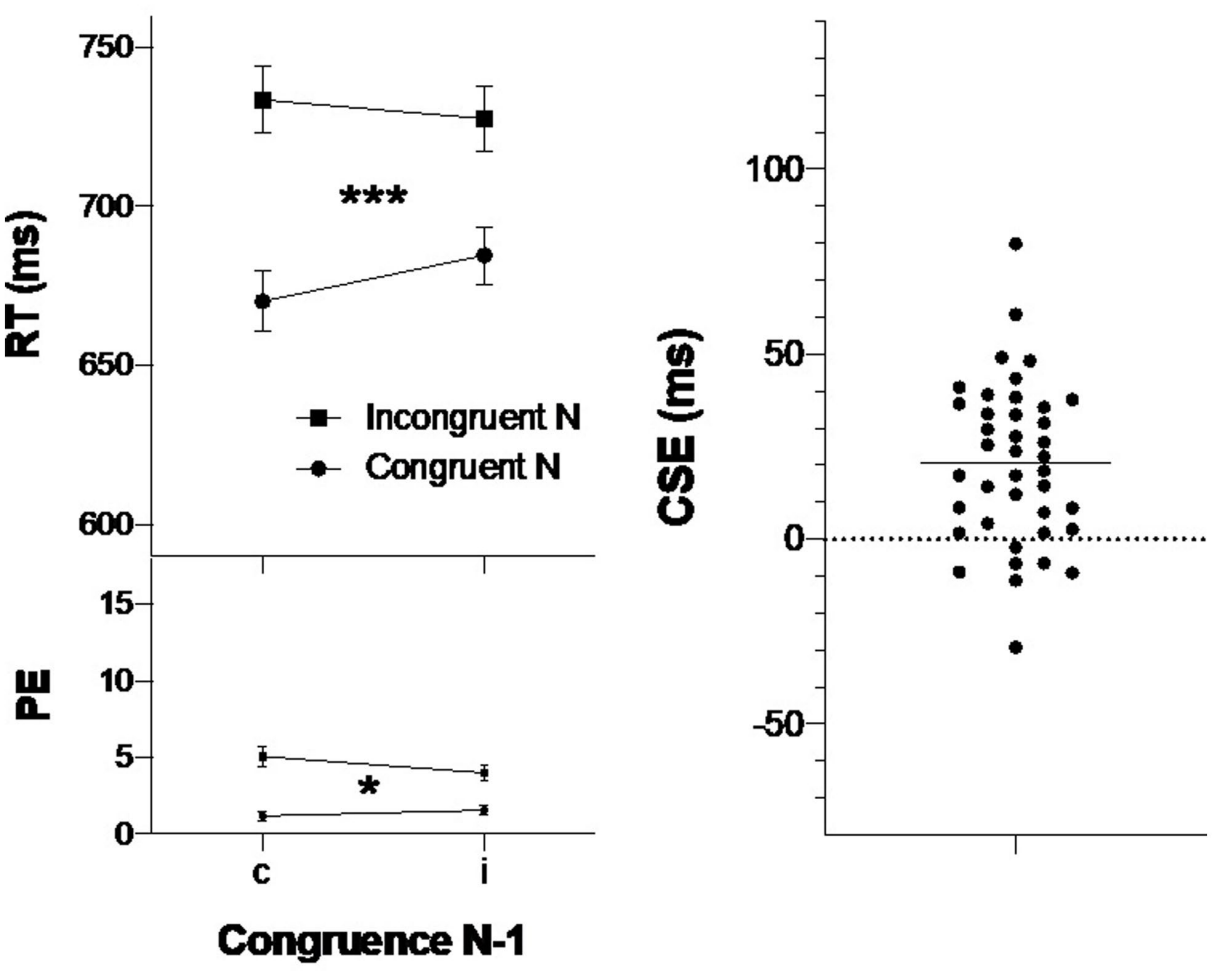
Response times (RT) and percent error (PE) in Experiment 3 as a function of previous and current congruency (left). The individual congruence sequence effects (CSE) are calculated as the congruence effect following congruent trials minus the congruence effect following incongruent trials. The sample mean is represented by the horizontal solid line (right). Error bars represent standard errors of the mean. ***p < 0.001, *p < 0.05.

The repeated measures ANOVA with the within-subject factors Congruence_N_ and Congruence_N-1_ was conducted on RTs and percent error (PE). For all analyses, the first trial of each block, post-error trials, and trials in which RTs exceeded the response deadline (missed trials) were excluded (4.8%). For the RT analysis, incorrect trials (3.0%) and trials with RTs smaller or larger than 3 SD of the individual condition mean (1.6%) were removed. One-sided paired t-tests were run to test for the influence of previous (non)conflict trials on current incongruent trials and on current congruent trials. Results are presented in Figure 4.

#### RT

The factor Congruence_N_ was highly significant, *F*(1, 39) = 146.22, *p* < 0.001, η_p_^2^ = 0.79, with smaller RTs in congruent (677 ms) than in incongruent (731 ms) trials. The effect of previous congruence slightly missed the significance level, *F*(1, 39) = 3.95, *p* = 0.054, η_p_^2^ = 0.09. Importantly, the factors Congruence_N-1_ and Congruence_N_ showed a strong interaction, *F*(1, 39) = 34.49, *p* < 0.001, η_p_^2^ = 0.47. Subsequent t-tests confirmed a significant reduction of RTs in incongruent trials, *t*(39) = 2.00, *p* = 0.026 (one-sided), and a significant increase of RTs in congruent trials *t*(39) = −5.70, *p* < 0.001 (one-sided), following conflict compared to previous non-conflict trials. A follow-up exploratory analysis with the additional 8-level factor Inter-trial-interval did not modulate the CSE, *F*(7, 273) = 1.40, *p* = 0.227, η_p_^2^ = 0.04. Also, the factor Response repetition (repetition versus alternation) had no impact on the CSE, *F*(1, 39) = 2.53, *p* = 0.120, η_p_^2^ = 0.06.

#### PE

Error rates mirrored the RT result pattern. A strong congruence effect was expressed in less erroneous responses in congruent (1.2%) than in incongruent trials (4.5%), *F*(1, 39) = 39.84, *p* < 0.001, η_p_^2^ = 0.51. There was no effect of Congruence_N-1_, *F*(1, 39) = 1.20, *p* = 0.280, η_p_^2^ = 0.03, but an interaction between both factors, *F*(1, 39) = 5.72, *p* = 0.022, η_p_^2^ = 0.13, indicating a CSE. Subsequent t-tests showed that conflict (compared to no conflict) reduced error rates in subsequent incongruent trials, *t*(39) = 2.07, *p* = 0.023 (one-sided). There was no significant conflict modulation in congruent trials, *t*(39) = −1.15, *p* = 0.128 (one-sided). As in RTs, the CSE did not depend on specific ITIs, *F*(7, 273) = 0.59, *p* = 0.761, η_p_^2^ = 0.02. Nor was the CSE modulated by the additional factor Response repetition (repetition vs. alternation), *F*(1, 39) = 0.61, *p* = 0.439, η_p_^2^ = 0.02.

### Discussion

Simplifying the confound-minimized design from Experiments 1 and 2 revealed a strong CSE pattern in both dependent measures. It demonstrates that the present prime-Stroop task is sufficient to produce sequential modulations of the conflict effect. Compared to our prior experiments with four response alternatives on the right hand, we now included a binary forced-choice RT task, categorizing all face stimuli as either male or female with left- and right-hand responses. Additionally, stimulus presentation time was increased to 1,000 ms, and the experiment was conducted in the lab. The CSE was not modulated by the duration of the inter-trial interval, nor by response repetitions or alternations.

## Experiment 4a and 4b

Based on the positive results of Experiment 3, Experiments 4a and 4b were set up to again implement a confound-minimized design. We decided to maintain the long stimulus presentations, as well as the left- and right-hand responses, because the index and middle fingers exhibit higher independent movement control than the ring and little finger (Lang and Schieber, 2004). Other than these changes, Experiment 4a largely mirrored Experiment 1. Experiment 4b was run in parallel and served as a replication and extension of Experiment 4a using an adapted design more suitable for potential fMRI application. In particular, we excluded the two shortest ITIs to accommodate the temporal BOLD dynamics. In addition, target stimulus presentation was not terminated with response execution but remained on screen for the full 1,000 ms instead to ensure constant visual presentation for all trial types.

## Experiment 4a

### Methods

#### Participants

A total of 40 students from the University of Greifswald (34 female; *M*_age_ = 24.5 years, SD = 4.7 years, range 19–37 years) participated in the online experiment and received course credits or €5. All participants had normal or corrected-to-normal vision, and all but two reported being right-handed, including one ambidextrous participant. None of the participants had a first name that was used as a stimulus in the study. Participants provided informed consent before their inclusion in the study in accordance with the ethical standards of the 1964 Declaration of Helsinki and of the German Psychological Society.

#### Stimuli and apparatus

The same face and name stimuli were used as in Experiments 1 and 3. Responses, however, were given with the left middle and index finger (Subset A) and with the right index and middle finger (Subset B). Response keys were A and S for the left hand and K and L for the right hand on the QWERTZ keyboard.

#### Procedure

**Figure 5:**
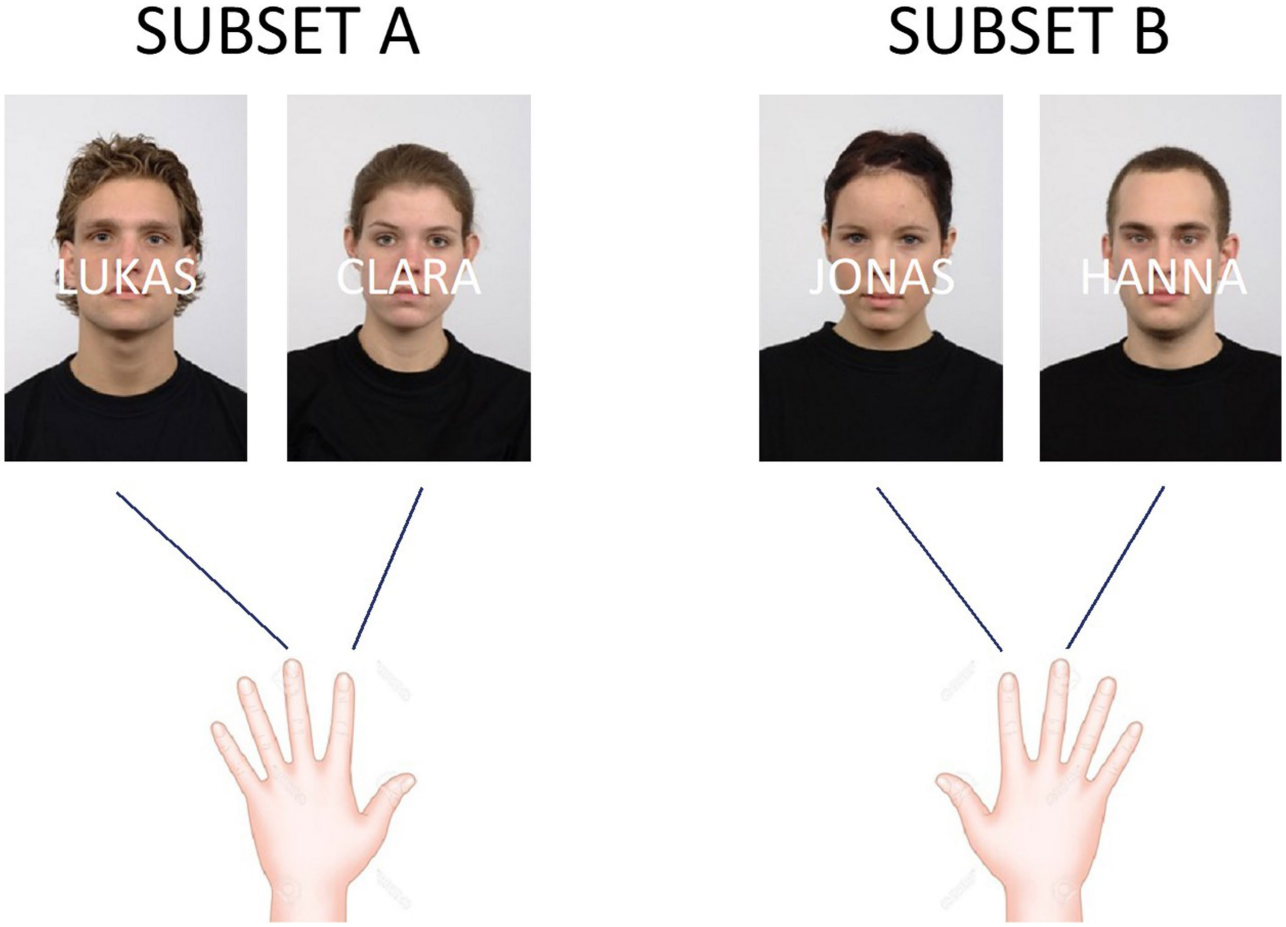
Two face and two name stimuli formed a subset of stimuli and responses. Incongruent face-name pairings occurred only within each subset. Congruent face-name pairings are shown for Subset A, and incongruent face-name pairings are shown for Subset B. Note that incongruent face-name pairings occurred exclusively within each subset. Stimuli and responses of Subset A and B are alternated on a trial-by-trial basis.

The experiment consisted of a learning and experimental phase, as in Experiments 1 and 2. The trial structure was the same as in Experiment 3. Participants responded using the index and middle fingers of both hands, with one hand assigned to Subset A and the other assigned to assigned to Subset B (see Figure 5). All participants responded to female face identities with their index fingers and to male face identities with their middle fingers. More specifically, half of the participants responded to Lukas and Clara with the left middle and index finger and to Hanna and Jonas with the right index and middle finger, respectively. The other half of the participants responded to Jonas and Hanna with the left middle and index finger and to Clara and Lukas with the right index and middle finger, respectively.

#### Design

A 2 (Congruence_N_: congruent vs. incongruent) × 2 (Congruence_N-1_: congruent vs. incongruent) within-subjects repeated measures design was applied.

### Results

**Figure 6:**
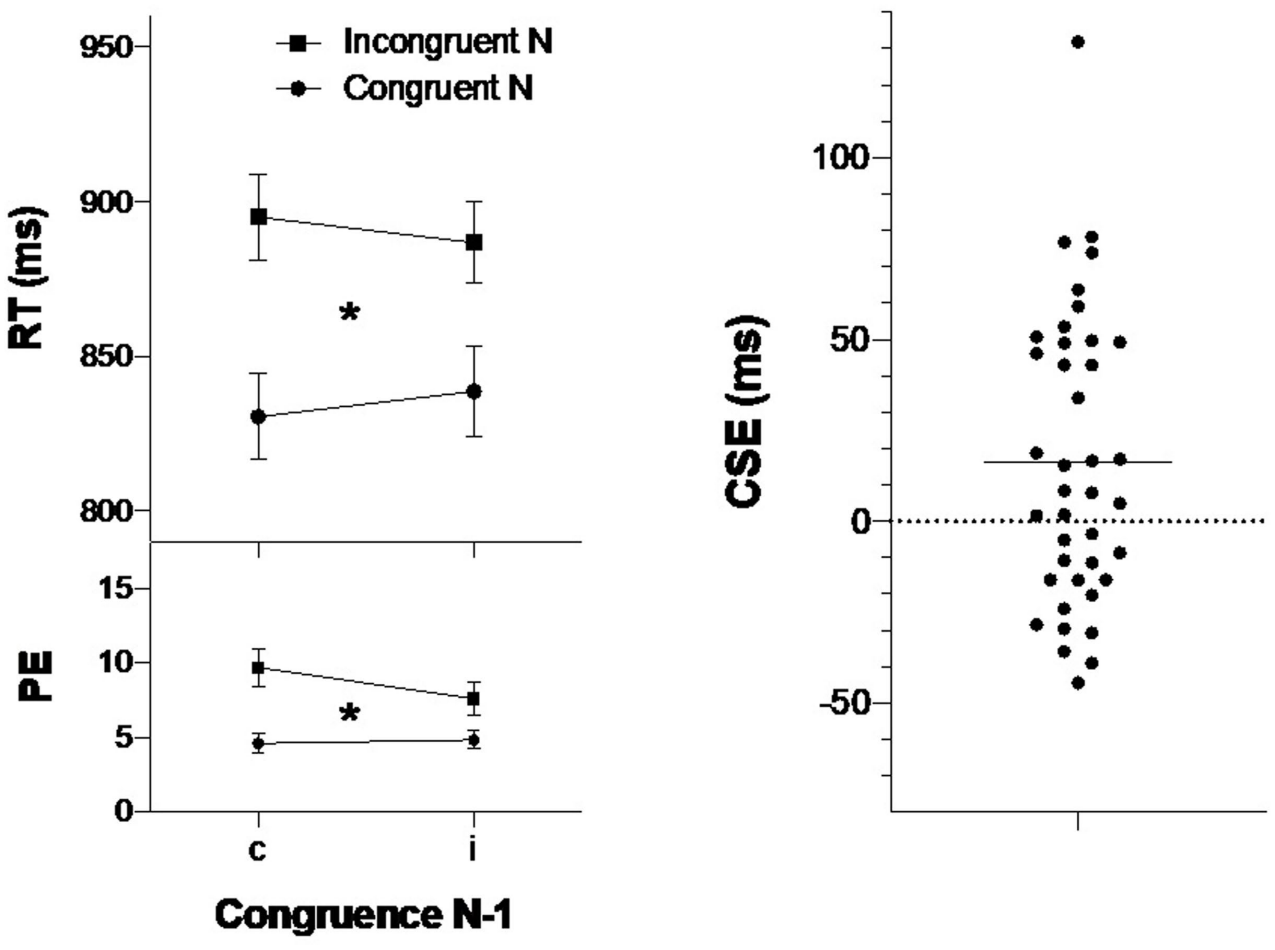
Response times (RT) and percent error (PE) in Experiment 4a are presented as a function of previous and current congruency (left). The individual congruence sequence effects (CSE) are calculated as the congruence effect following congruent trials minus the congruence effect following incongruent trials. The sample mean is represented by the horizontal line (right). Error bars represent standard errors of the mean. *p < 0.05.

*Data analysis.* The repeated measures ANOVA, including the within-subject factors Congruence_N_ and Congruence_N-1_ was conducted on RTs and percent error (PE). For all analyses, the first trial of each block, post-error trials, and trials in which RTs exceeded the response deadline (missed trials) were excluded (10.0%). For the RT analysis, incorrect trials (6.7%) and trials with RTs smaller or larger than 3 SD of the individual condition mean (1.5%) were removed. One-sided paired t-tests were run to test for the influence of previous (non)conflict trials on current incongruent trials and on current congruent trials. Results are presented in Figure 6.

#### RT

The repeated measures ANOVA on RTs confirmed a substantial congruence effect, *F*(1, 39) = 132.36, *p* < 0.001, η_p_^2^ = 0.77. Participants responded faster in congruent (835 ms) than in incongruent (891 ms) trials. Previous congruence did not affect current responses, as the factor Congruence_N-1_ was not significant, *F* < 1. Most importantly, there was a significant interaction between Congruence_N-1_ and Congruence_N_, *F*(1, 39) = 6.74, *p* = 0.013, η_p_^2^ = 0.15. Previous conflict (compared to previous non-conflict) slowed subsequent responses in congruent trials, *t*(39) = −2.11, *p* = 0.021 (one-sided), and led to faster responses in incongruent trials, *t*(39) = 1.71, *p* = 0.048 (one-sided). A follow-up exploratory analysis with the additional 8-level factor Inter-trial-interval did not modulate the CSE, *F*(7, 273) = 0.26, *p* = 0.970, η_p_^2^ = 0.01.

#### PE

The repeated measures ANOVA on error rates closely mirrored the RT results. There was a significant effect of Congruence_N_, *F*(1, 39) = 25.49, *p* < 0.001, η_p_^2^ = 0.40, with fewer errors in congruent (4.7%) than in incongruent trials (8.6%). The numerical difference in error rates between previous congruence (7.1%) and previous incongruence (6.2%) missed statistical significance, *F*(1, 39) = 3.91, *p* = 0.055, η_p_^2^ = 0.09. Finally, both factors interacted, *F*(1, 39) = 6.73, *p* = 0.013, η_p_^2^ = 0.15, demonstrating a CSE. Subsequent t-tests confirmed a strong decline in error rates for incongruent trials following previous incongruence, *t*(39) = 2.93, *p* = 0.003 (one-sided). The analogous increase in error rates for congruent trials following previous incongruence was not significant, *t*(38) = −0.43, *p* = 0.335 (one-sided).

## Experiment 4b

### Methods

#### Participants

A total of 40 students from the University of Greifswald (28 female; *M*_age_ = 23.0 years, SD = 5.1 years, range 18–40 years) participated in the online experiment and received either course credits or €5. All participants had normal or corrected-to-normal vision, and all but two reported being right-handed. None of the participants had a first name that was used as a stimulus in the study. Participants provided informed consent before their inclusion in the study in accordance with the ethical standards of the 1964 Declaration of Helsinki and of the German Psychological Society.

#### Stimuli, apparatus, and procedure

Experiment 4b was virtually identical to Experiment 4a except for the following changes: Using the same stimulus subsets as before, stimulus–response mappings were now counterbalanced, but gender was constant for homologous response fingers. More precisely, when Lukas (Subset A) was responded to with either the left index or left middle finger (counterbalanced between participants), Jonas (Subset B) was responded to with the corresponding right index or right middle finger, respectively. The same also applied when responding to female faces. In Experiment 4b, the two shortest ITIs (1,000 and 1,500 ms) were omitted. Furthermore, target stimuli were presented for 1,000 ms, and thus, response execution did not offset the target stimulus presentation.

#### Design

A 2 (Congruence_N_: congruent vs. incongruent) × 2 (Congruence_N-1_: congruent vs. incongruent) within-subjects repeated measures design was applied.

### Results

#### Data analysis

**Figure 7:**
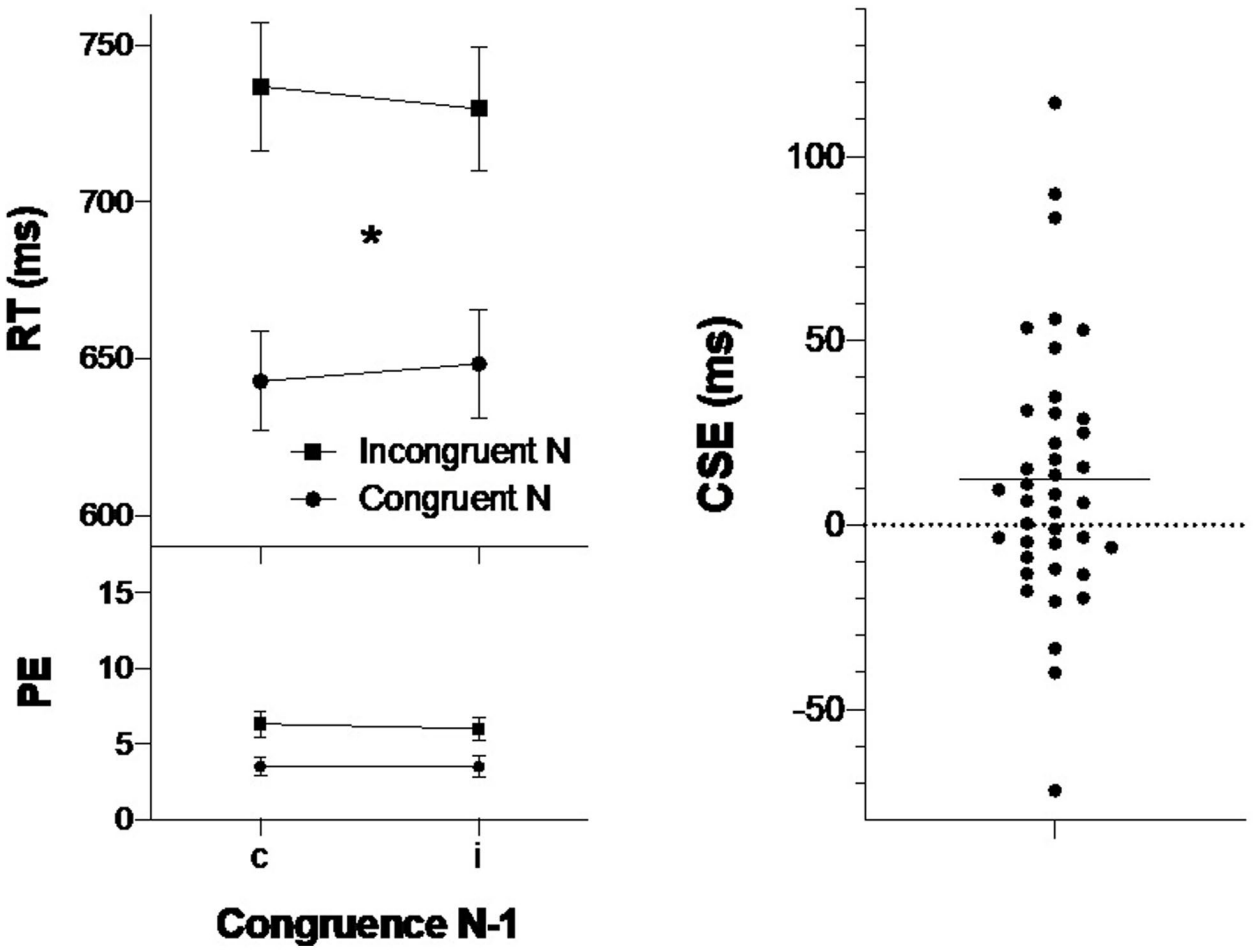
Response times (RT) and percent error (PE) in Experiment 4b are presented as a function of previous and current congruency (left). The individual congruence sequence effects (CSE) are calculated as the congruence effect following congruent trials minus the congruence effect following incongruent trials. The sample mean is represented by the horizontal line (right). Error bars represent standard errors of the mean. *p < 0.05.

The repeated measures ANOVA, including the within-subject factors Congruence_N_ and Congruence_N-1,_ was conducted on RTs and percent error (PE). For all analyses, the first trial of each block, post-error trials, and trials in which RTs exceeded the response deadline (missed trials) were excluded (7.6%). For the RT analysis, incorrect trials (4.8%) and trials with RTs smaller or larger than 3 SD of the individual condition mean (1.6%) were removed. One-sided paired t-tests were run to test for the influence of previous (non)conflict trials on current incongruent trials and on current incongruent trials and on current congruent trials. Results are presented in Figure 7.

#### RT

The repeated measures ANOVA on RTs revealed a significant main effect of the factor Congruence_N_, *F*(1, 39) = 148.17, *p* < 0.001, η_p_^2^ = 0.79. RTs in congruent conditions were considerably shorter (643 ms) than RTs in incongruent conditions (733 ms). While the factor Congruence_N-1_ did not turn significant, *F*(1, 39) = 0.08, *p* = 0.778, η_p_^2^ = 0.00, the interaction between both factors did, *F*(1, 39) = 5.07, *p* = 0.030, η_p_^2^ = 0.12. Subsequent t-tests showed a slight reduction of incongruent RTs and a slight increase of congruent RTs following conflict, both of which, however missed statistical significance, *t*(39) = 1.60, *p* = 0.059 (one-sided) and *t*(39) = −1.58, *p* = 0.062 (one-sided), respectively. A follow-up exploratory analysis with the additional 6-level factor Inter-trial-interval did not modulate the CSE, *F*(5, 195) = 0.55, *p* = 0.736, η_p_^2^ = 0.01.

#### PE

Participants committed less errors in congruent (3.5%) than in incongruent trials (6.2%), *F*(1, 39) = 22.59, *p* < 0.001, η_p_^2^ = 0.37. Previous congruence did not affect current error rates, *F*(1, 39) = 0.25, *p* = 0.622, η_p_^2^ = 0.01. Also, both factors did not interact, *F*(1, 39) = 0.09, *p* = 0.763, η_p_^2^ = 0.00.

#### Between-experiment comparison

An analysis of the combined data sets with N = 80 and Experiment as a between-subject factor was run. For RTs, there was a significant difference in overall response speed between the two experiments, *F*(1, 78) = 59.34, *p* < 0.001, η_p_^2^ = 0.43. Participants were much faster in Experiment 4b (689 ms) than in Experiment 4a (863 ms). In addition, the congruence effect was more pronounced in Experiment 4b (88 ms) than in Experiment 4a (56 ms), *F*(1, 39) = 13.01, *p* < 0.001, η_p_^2^ = 0.14. The CSE, *F*(1, 78) = 11.81, *p* < 0.001, η_p_^2^ = 0.13, however, did not differ, *F*(1, 78) = 0.20, *p* = 0.656, η_p_^2^ = 0.00. For error rates, the congruence effect, *F*(1, 78) = 47.29, *p* < 0.001, η_p_^2^ = 0.38, was similar in both experiments, *F*(1, 78) = 1.70, *p* = 0.196, η_p_^2^ = 0.02. The interaction between Congruence_N-1_ and Congruence_N_ remained significant, *F*(1, 78) = 4.08, *p* = 0.047, η_p_^2^ = 0.05, and did not statistically differ between experiments, *F*(1, 91) = 2.50, *p* = 0.118, η_p_^2^ = 0.03.

#### Effect variability

Inspection of the data across the five experiments suggested substantial inter-individual variability in the CSE. A Levene’s test confirmed that variability differed across experiments, *F*(4, 208) = 3.74, *p* = 0.006, with Experiment 3 showing roughly half the variability of the other experiments (see Table 1).

### Discussion

Experiments 4a and 4b pursued two aims. First, Experiment 4a aimed to extend the observed CSE in Experiment 3, which used two response hands, by employing a confound-minimized design. Second, Experiment 4b served to extend this aim and replicate this CSE, including further changes to optimize the paradigm for potential use in an fMRI context. Importantly, small but reliable CSE’s were found in both experiments.

## General discussion

fMRI research frequently employs response conflict tasks, such as the Stroop task, to gain insight into the neural mechanisms underlying executive control and the adaptation to conflict (Milham et al., 2002; Polk et al., 2008; Parris et al., 2019; Okayasu et al., 2023). Especially the face-word version of the Stroop task has become popular in neuroimaging (Ovaysikia et al., 2011; Kar et al., 2019; Oren et al., 2023) because the separation of sensory regions for relevant and irrelevant processing allows for a better understanding of the neural mechanisms of cognitive control (Egner and Hirsch, 2005; Ehlis et al., 2024). However, studying how the brain adapts to ongoing conflict using standard response conflict tasks in fMRI settings presents some specific challenges. In a set of five experiments, the present study aimed to address two key methodological issues: Verifying that the observed trial-by-trial adjustments are genuinely driven by cognitive control processes while ensuring the temporal stability of the behavioral marker of conflict adaptation across varying inter-trial intervals.

Regarding the first aim, a confound-minimized version of a face-word Stroop task was designed (Experiment 1) to eliminate potential influences of stimulus and response repetitions, ensuring that the CSE can be interpreted as an adaptation of cognitive control in response to conflict. For this, we used two 2-alternative forced-choice tasks that consisted of separate stimulus and response sets, and alternated from trial to trial. Moreover, to avoid contingency learning, each stimulus served equally often as a congruent or an incongruent stimulus. To further increase the involvement of cognitive control, the irrelevant name was presented with a head start prior to the target stimulus (Weissman et al., 2014; Weissman et al., 2015b). Regarding the second aim, all experiments included a wide range of inter-trial intervals (up to 5 s) to accommodate the temporal requirements when measuring the BOLD signal in an imaging setting.

In the first two experiments, participants responded with four fingers of their right hand either to the learned identity of a person’s face (Experiment 1) or a celebrity’s face (Experiment 2) while ignoring his or her name printed on top. While substantial Stroop interference of about 50 ms was observed in both experiments, no evidence of conflict adaptation, as expressed in a non-significant CSE, was found. Experiment 3 used a simplified gender categorization Stroop task (Egner et al., 2008). The presentation of the target faces was extended to 1,000 ms, and participants responded with the left and right index fingers. Here, Stroop interference was of a similar size as in Experiments 1 and 2, but now a strong trial-by-trial modulation of the Stroop effect (i.e., a CSE) was observed. Response repetitions or alternations, as typically involved in a binary choice task, did not affect the CSE. Experiments 4a and 4b maintained the prolonged target presentation of Experiment 3 and applied the confound-minimization protocol of Experiments 1 and 2. However, participants now responded to the identity of four faces with the index and middle fingers of the left and right hands. In both experiments significant CSEs were obtained. In none of the experiments reporting CSEs did the inter-trial interval influence the CSE pattern.

Together, the present results show that a strong pattern of a CSE can be demonstrated in a classical gender categorization face-word Stroop task (Experiment 3), replicating numerous previous studies (Egner et al., 2008, 2010; Jiménez et al., 2020; Weissman and Carp, 2013). Second, controlling for repetition effects and applying a confound-minimized face-word Stroop task with variable inter-trial intervals appeared to restrict the observation of a CSE to certain conditions, namely the use of two response hands and sufficiently long target stimulus presentation (Experiment 4a and 4b). However, in both of these experiments, the CSE was found despite a trial-by-trial alternation of left and right response hands. Whereas previous research has shown a substantial reduction of CSEs when effector systems are alternated (e.g., manual vs. foot responses; Janczyk and Leuthold, 2018), the present findings indicate that alternating effectors within the same response modality, i.e., switching between two hands, did not eliminate the CSE (see also Weissman et al., 2015a).

At present, however, it remains unclear to what extent the longer target presentation time, the binary response hand mapping, or both contributed to the emergence of the CSE in Experiment 4a and 4b. Longer target presentation times may facilitate conflict processing, and responses executed with the index and middle fingers of both hands may allow for more independent motor control than responses involving the ring and little fingers (Lang and Schieber, 2004). However, CSEs in confound-minimized priming tasks have also been demonstrated with target presentations shorter than 200 ms and with four responses mapped onto a single hand (Schiltenwolf et al., 2023). Thus, further research will be required to disentangle the relative contribution of stimulus presentation time and response mapping to the emergence of the CSE.

Finally, the CSE in our face-word Stroop tasks was not influenced by variable inter-trial intervals of up to 5 s, replicating recent findings of a confound-minimized priming task (Schiltenwolf et al., 2023). Schiltenwolf et al. argued that the exclusion of feature repetitions in a confound-minimized design reduces binding effects that are subject to decay. However, a lack of interaction between the CSE and inter-trial interval was also observed in our binary gender categorization face-word Stroop task in Experiment 3, which was not confound minimized. A stable CSE across long temporal intervals is at odds with reports of declining CSEs with increasing inter-trial intervals obtained in similar tasks (Duthoo et al., 2014a; Egner et al., 2010). This might be reconciled with the findings of Duthoo et al. (2014b), arguing that a stronger involvement of proactive control can prevent the CSE from decaying. Whereas Duthoo et al. (2014b) (Experiment 2) increased the proportion of long inter-trial intervals to enhance proactive control, the inclusion of a priming component in Schiltenwolf et al. (2023) and in the present experiments may have achieved a similar effect.

Overall, the present findings demonstrate that a reliable CSE can be obtained in a confound-minimized face-word Stroop task, including long inter-trial intervals. This enables a cognitive control-based interpretation of the CSE and allows for an application of studying cognitive control adjustments in imaging contexts through temporal trial jittering. However, this methodological rigor appears to come with a significant cost: improvements that enhance experimental precision and interpretability also increase paradigm complexity and tend to produce smaller effect sizes accompanied by substantial inter-individual variability. That is, the inter-individual variability of the CSE was much less pronounced in a setting that allowed stimulus and response repetitions and included a reduced response set with 2 response alternatives (Experiment 3). Given that the increased stimulus presentation time was the same between Experiments 3 and 4a/b, it seems that the reduced experimental complexity in Experiment 3 is the driving factor for larger and stable CSEs (e.g., Blais and Verguts, 2012; Lee et al., 2025).

Thus, researchers are faced with the need to find a balance between obtaining sufficiently large effect sizes and achieving maximal experimental control. Aiming for large effect sizes may mean accepting potential contributions of additional, non–control-based influences on the CSE. Maximizing experimental control ensures cleaner measures of control processes but is often accompanied by increased inter-individual variability in behavioral outcomes. Consequently, assessing intra-individual stability of the behavioral effects is essential. Although recent work on the CSE using a variant of a face-word Stroop task has reported low test–retest reliability (Schuch et al., 2022), corresponding evidence for the present paradigms is still lacking. The challenge of high inter-individual variability is also evident in neuroimaging research, where typical group-averaged results may conceal individual neural activity patterns. However, recent developments in precision neuroimaging procedures aim to reduce the frequently observed inter-individual heterogeneity by sampling several hours of data for individual participants (Gratton et al., 2020; Michon et al., 2022). This methodology has shown promise to elicit more consistent neural activity during cognitive control tasks within individuals, albeit with substantial between-subject variability (Michon et al., 2025). Hence, such an approach may be promising for studying the neural mechanisms of cognitive control in health and disease (e.g., Hartman et al., 2025).

## Limitations

While the present study aimed to examine the temporal stability of the CSE across varying ITIs in a confound-minimized design and to evaluate its potential suitability for paradigms involving temporal jittering in fMRI research, a few limitations of the present study should be noted. First, the ITIs used in the present experiments were still relatively short. That is, intervals of 2 s or less would generally not be suitable for fMRI applications, and only Experiment 4b implemented ITIs approaching common minimum recommendations. Furthermore, although increased methodological rigor in confound-minimized designs enhances experimental control and interpretability, they tend to produce smaller effect sizes accompanied by substantial inter-individual variability. Accordingly, the present results should be interpreted with caution when considering direct translation to fMRI research. Finally, although we controlled for major experimental confounds when assessing the CSE in a face-word Stroop paradigm, we did not account for single-subject stimulus salience, as we used names that may be of personal significance to the participants. Encountering a significant other’s name (e.g., Lukas, a brother’s name) may not only affect cognitive processing but also modulate neural activity in self-referential and attentional networks (Tacikowski et al., 2011) and could affect the neural activation patterns of the CSE. Thus, future studies could adapt strategies to diminish name associations to ensure equal stimulus processing across participants.

## Conclusion

Across the five experiments, we demonstrated how a confound-minimized face-word Stroop paradigm can be optimized to isolate control-based CSE that is temporally stable. We incorporated elements that enhance fMRI compatibility by applying jittered ITIs, an adaptable manual response setup, and the use of stimuli whose neural processing of task (ir)relevant features is dissociable. Yet, translating behavioral advances into neuroimaging results requires careful consideration of study-specific requirements, as methodological rigor was accompanied by high inter-individual variability. These limitations could be improved by investigating the intra-individual stability of behavioral markers and by considering new developments in high-precision neuroimaging procedures.

## Data Availability

The raw data supporting the conclusions of this article are available. DOI: 10.23668/psycharchives.21818.

## Footnotes

1
https://pavlovia.org
